# Understanding adsorption geometry of organometallic molecules on graphite

**DOI:** 10.1038/s41598-021-97978-x

**Published:** 2021-09-16

**Authors:** Seungtaek Oh, Jungyoon Seo, Giheon Choi, Hwa Sung Lee

**Affiliations:** 1 Department of Materials Science and Chemical Engineering, Hanyang University, Ansan, Gyeonggi 15588 Republic of Korea; 2 BK21 FOUR ERICA-ACE Center, Hanyang University, Ansan, Gyeonggi 15588 Republic of Korea

**Keywords:** Chemistry, Engineering, Materials science

## Abstract

To comprehensively investigate the adsorption geometries of organometallic molecules on graphene, Cp^*^Ru^+^ fragments as an organometallic molecule is bound on highly oriented pyrolytic graphite and imaged at atomic resolution using scanning tunneling microscopy (STM) (Cp^*^ = pentamethylcyclopentadienyl). Atomic resolution imaging through STM shows that the Cp^*^Ru^+^ fragments are localized above the hollow position of the hexagonal structure, and that the first graphene layer adsorbed with the fragments on the graphite redeveloped morphologically to minimize its geometric energy. For a better understanding of the adsorption site and molecular geometry, experimental results are compared with computed calculations for the graphene surface with Cp^*^Ru^+^ fragments. These calculations show the adsorption geometries of the fragment on the graphene surface and the relationship between the geometric energy and molecular configuration. Our results provide the chemical anchoring geometry of molecules on the graphene surface, thereby imparting the theoretical background necessary for controlling the various properties of graphene in the future.

## Introduction

Recently, graphene with highly ordered carbon nanostructures has garnered significant interest as a smart material with high electrical and thermal conductivities and excellent mechanical properties^1–5^. Bandgap control in graphene is one of the most important and tantalizing research topics in the graphene community because it may ultimately enable new applications in digital electronics^6,7^, pseudospintronics^8^, terahertz technology^9,10^, and infrared nanophotonics^11,12^. A number of approaches have been proposed or implemented to control the bandgap in graphene, such as using uniaxial strain^13,14^, graphene–substrate interactions^15,16^, lateral confinement^17^, and breaking the inversion symmetry in bilayer graphene^18^. Among them, adhering or bonding the atoms/molecules on the graphene surface can effectively control the bandgap because of the large surface-to-volume ratio of atoms/molecules that can be easily adsorbed on its surface^19,20^. However, comprehensive investigations into the adsorption behavior of molecules on graphene surfaces are insufficient, although the understanding and interpretation of the molecule–graphene interaction is a fundamental research field.

As a model to investigate the adsorption behavior of molecules on graphene, organometallic compounds were considered in this study because of their diverse applications in surface science and nanotechnology, such as catalysis^21^, tribology^22^, molecular electronics^23^, and molecular magnetism^24,25^. In particular, an organometallic molecule containing the Cp^*^Ru^+^ fragment (Cp^*^ = pentamethylcyclopentadienyl) is ubiquitous in complex chemistry and their thermal and photochemical behaviors as well as reactive intermediates for selective reactions have garnered significant attention^26–28^. The Cp^*^Ru^+^-graphene complex can be obtained via the reaction between Cp^*^Ru(CH_3_CN)_3_PF_6_ and arenes, in which the relatively labile acetonitrile ligands are readily substituted by a 6π-electron donor^29^. To analyze individual Cp^*^Ru^+^ fragments adsorbed on arene structures, it is important to select graphene substrates that are highly uniform over a wide area. Hence, highly oriented pyrolytic graphite (HOPG) was selected in this study to model an atomically perfect graphene surface using the mechanical exfoliation method.

We investigated the adsorption behavior of Cp^*^Ru^+^ fragments on a graphene surface exfoliated from high-quality HOPG surfaces. The purpose of this study was to comprehensively investigate the identification of individual organometallic adsorbates via specialized local measurements and then to test the design criteria for adsorbing an organometallic compound on graphene. The advent of surface probing techniques such as atomic force microscopy (AFM) and scanning tunneling microscopy (STM) has resulted in increasing interest in the study of the adsorption behavior and geometric configuration of individual molecules on substrate surfaces. Furthermore, a comprehensive theoretical geometry of the Cp^*^Ru^+^ fragment on the graphene surface was developed by performing computational functions of molecular mechanics calculations.

## Experimental

### Sample preparation

Pentamethylcyclopentadienyltris(acetonitrile)ruthenium(II) hexafluorophosphate [Cp^*^Ru(CH_3_CN)_3_PF_6_] and HOPG with ZYH grade were purchased from Aldrich Chem. and Advanced Ceramics Corp., respectively. To prepare Cp^*^Ru^+^-graphite, the HOPG was mechanically cleaved using 3M scotch tape in air and immediately dipped into a Cp^*^Ru(CH_3_CN)_3_PF_6_ solution based on ethanol at room temperature. After the reaction or prior to the characterization, the HOPG surface was sufficiently washed with copious amounts of ethanol and distilled water and then dried by blowing N_2_ gas.

### Characterization

The presence of the Cp^*^Ru^+^ fragment was characterized via Fourier transform infrared spectroscopy (FT-IR, Perkin Elmer System 2000) and X-ray photoelectron spectroscopy (XPS). The XPS spectra were recorded on a VG ESCALAB 220i spectrometer using Mg Kα radiation (1253.6 eV), which was operated at 15 kV and 20 mA. To exclude the effect of [Cp^*^Ru(CH_3_CN)_3_]^+^ residue on the substrate, the substrate was washed with copious amounts of ethanol prior to the measurements. To visualize the Cp^*^Ru^+^ fragments on the HOPG surface, we used an atomic force microscope (Digital Instruments NanoScope III) and a scanning tunneling microscope (Digital Instruments NanoScope III). PtIr tips were used in the STM measurements. During the STM measurement, an insulating liquid, 1-phenyloctane (Aldrich Chem.) was used between the tip and the surface to allow high-quality atomic-scale imaging. During operation, the bias voltages and tunneling currents were varied from -10 to -500 mV and from 50 pA to 1 nA, respectively, to obtain high-quality images. A confocal backscattering Raman spectrum with a spot size of approximately 4 μm^2^ was measured using a 40X objective focused through the cell window. Furthermore, 3.2 mW of 633 nm He–Ne laser was used as the excitation source.

### Geometry calculation

The geometric configurations of Cp^*^Ru^+^-graphite were calculated from the molecular mechanics force field using the Polak–Ribiere algorithm in HyperChem Professional 8.0. To realize the graphite structure in the program, we used a five-layer graphene structure with 18 × 16 unit cells for each graphene (i.e., 576 carbon atoms).

## Results and discussion

**Figure 1:**
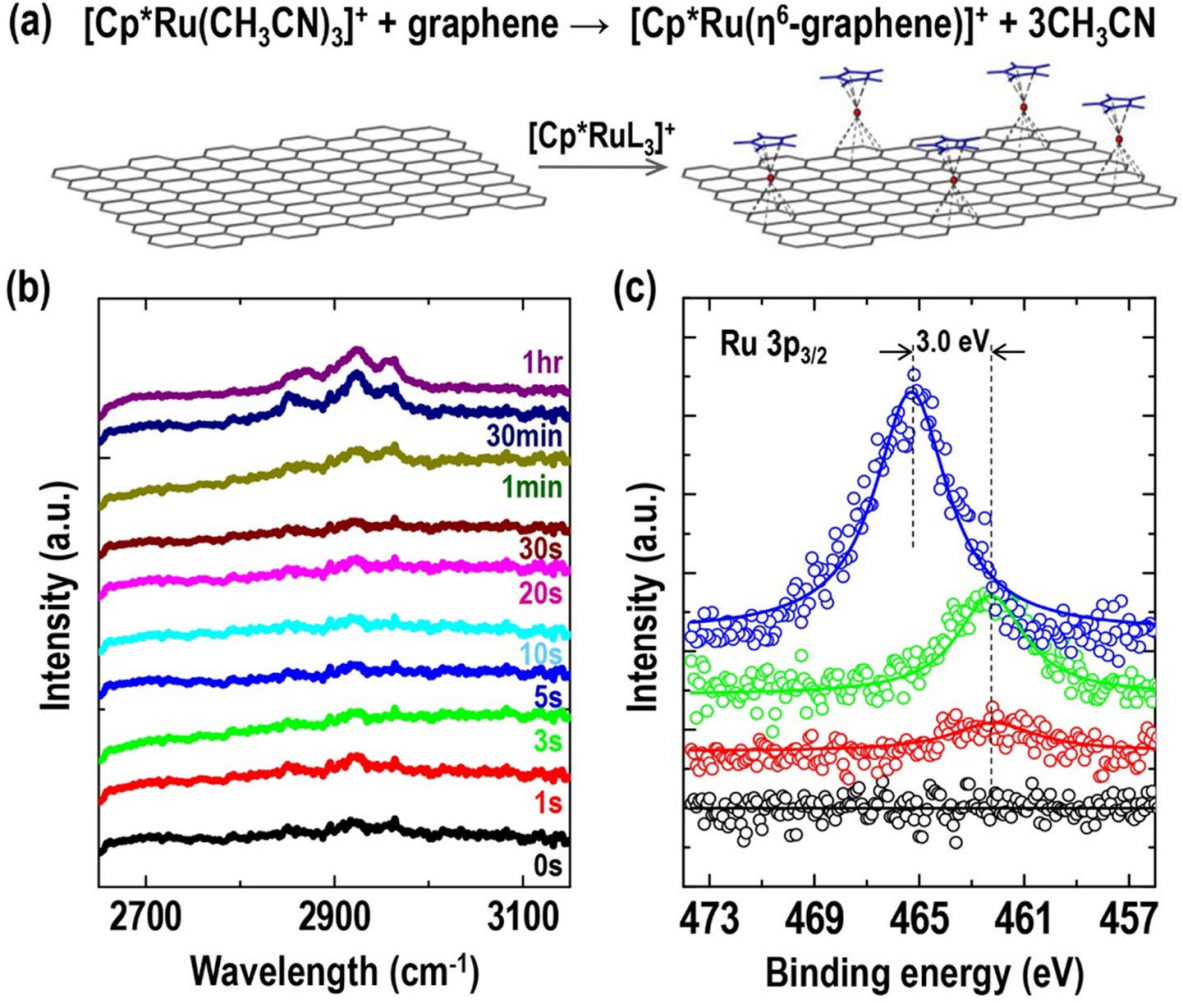
(**a**) Schematic illustration of chemical reaction between [Cp^*^Ru(CH_3_CN)_3_]^+^ and graphene surface. (**b**) FT-IR spectra of graphite as a function of reaction time in 1.25 mM Cp^*^RuL_3_PF_6_ solution. (**c**) XPS spectra of Ru 3p_3/2_ signal regions for pristine graphite (black), Cp^*^Ru^+^-graphites reacted with 1.25 × 10^−7^ M (red) and 1.25 mM (green) solutions, and dried 1.25 × 10^−7^ M Cp^*^RuL_3_PF_6_ on glass (blue).

To obtain an atomically flat graphene surface that exhibits a perfect lattice of thousands of angstroms, HOPG was used by applying the mechanical exfoliation method. [Cp^*^Ru(CH_3_CN)_3_]^+^ reacts readily with a various types of arenes to form *η*^6^-bound metal-arene complexes by coordination covalent bond, as shown in Fig. 1a. To adsorb Cp^*^Ru^+^ fragments on the graphene surface, we used the dipping method. The presence of Cp^*^Ru^+^ fragments on the graphene substrate was verified via FT-IR and XPS. Figure 1b shows the FT-IR spectra as a function of the reaction time. The pristine graphene substrate exfoliated from HOPG showed no indication of CH_3_-related peaks. As the reaction time progressed, the bands at 2922 and 2870 cm^−1^, assigned to the asymmetric and symmetric methyl (CH_3_) stretching modes of the methyl groups in Cp^*^, respectively^30,31^, increased sequentially. These results show that the Cp^*^ fragments originating from Cp^*^Ru^+^ reacted to the graphene surface as the reaction time progressed. Other verifications of the Cp^*^Ru^+^ fragments bound on the graphene surface can be confirmed through changes in Ru conditions, derived from XPS analysis. As shown by the XPS results in Fig. 1c, the peak at 465.3 eV corresponding to Ru 3p_3/2_ was observed in the case of dried Cp^*^Ru(CH_3_CN)_3_PF_6_ solution on the glass substrate (blue). Meanwhile, the presence of Ru 3p_3/2_in the dropped Cp^*^RuL_3_PF_6_ solution on the graphene (or HOPG substrate), was confirmed by the peaks at 462.3 eV, which induced a coordinative reaction between the Cp^*^Ru^+^ fragments and the graphene surface (green and red)^32–34^. This peak shift is important for explaining the reaction between Cp^*^Ru^+^ and the HOPG surface. The Ru^+^ atoms in [Cp^*^Ru(CH_3_CN)_3_]^+^ and Cp^*^Ru^+^-graphene have different atomic environments. In particular, the Ru^+^ in [Cp^*^Ru(CH_3_CN)_3_]^+^ is bound to acetonitrile ligands with relatively stronger electronegativity compared with the case involving reaction with the arene structure, where electrons (or the electron density) were attracted toward itself in a bond. Before discussing our results, it is noteworthy that the chemical shifts in the core-level binding energy of XPS are often used to investigate the electronic redistribution or charge transfer upon elements^35–37^. The general rule in the interpretation is that the binding energy of the atom increases with the electronegativity of the attached atoms or groups^35–37^. In other words, the Ru 3p_3/2_ peak in [Cp^*^Ru(CH_3_CN)_3_]^+^ shifts toward a higher binding energy compared with that in Cp^*^Ru^+^-graphite, as indicated by the XPS results shown in Fig. 1c. Based on the FT-IR and XPS results, it is clear that the Cp^*^Ru^+^ fragments were successfully bound on the graphene surface to form *η*^6^-bound metal-arene complexes by a coordination covalent bond during dipping the HOPG substrate in the [Cp^*^Ru(CH_3_CN)_3_]^+^ solution.

**Figure 2:**
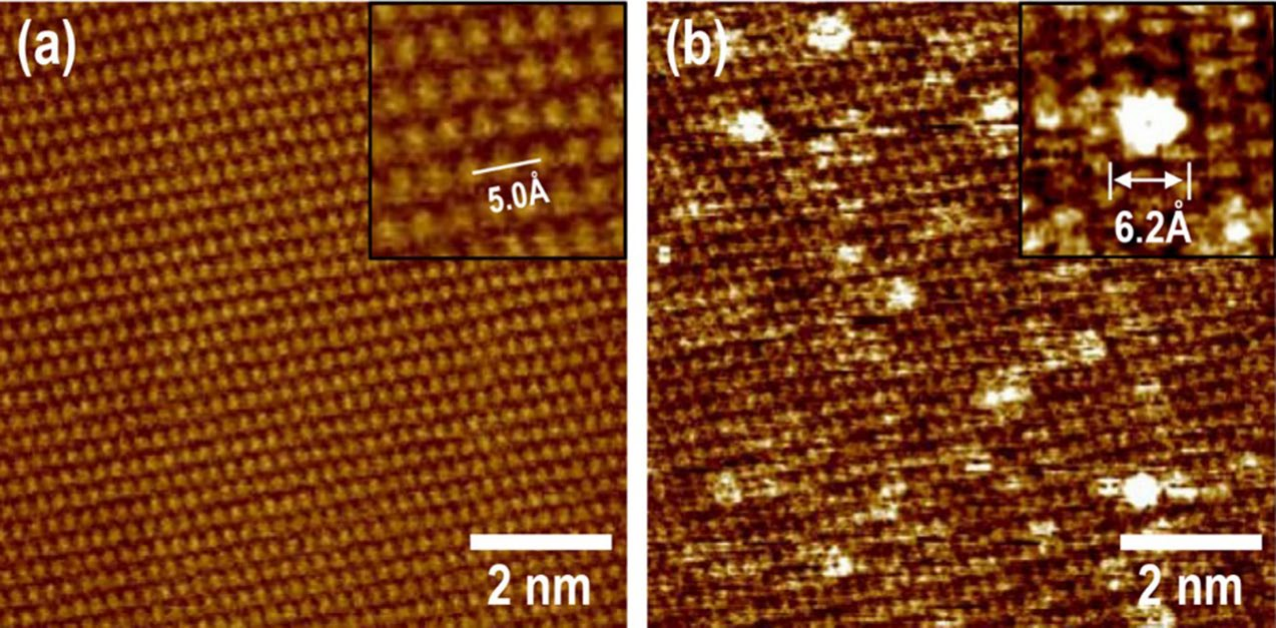
STM images of (**a**) pristine and (**b**) Cp^*^Ru^+^-graphites. Insets in (**a**) and (**b**) show enlarged images of hexagonal lattice structure of graphite and Cp^*^Ru^+^-fragment bound on graphite lattice structure, respectively. Cp^*^Ru^+^-graphite samples used in our STM experiments were prepared via reaction in 1.25 × 10^−15^ M solution for 10 s.

Confirming the adsorption sites of the individual molecules is crucial for understanding the anchoring geometry on the graphene surface. STM, which is one of the most sophisticated techniques for analyzing two-dimensional structural properties, provides detailed high-resolution atomic and molecular information. Furthermore, these studies have been additionally gaining momentum by combining the molecular simulations for individual systems. Figure 2a shows an atomic-resolution STM image of a pristine graphene surface, obtained by inserting an insulating liquid (1-phenyloctane) between the tip and surface, at a bias voltage of − 50 mV and a set point current of 100 pA. (In practice, we used a HOPG surface instead of graphene.) On the graphite surface layer, two types of carbon atoms with nonequivalent types existed: *α*- and *β*-site carbons^38^. The *α*-site carbon atoms in hexagonal graphite with ABAB stacking had neighbors directly below the second layer, whereas the *β*-site atoms were located above the hollow site of the layer beneath. These differences were attributed to the asymmetry of the interlayer interaction between the top layer and the layer located directly below or the structural site asymmetry of the hexagonal graphite. Such asymmetry induced differences in the local density of states as a consequence of the resulting interlayer interactions; hence, they were detected via STM. According to previous literature^38^, *β*-site carbons are visible as bright spots in STM images. In our STM images, we observed a hexagonal lattice structure with a distance of 2.48 Å between the tops of bright spots corresponding to the *β*-site atoms, although the effect of drift distortion on the image was observed, as shown in Fig. 2a. Figure 2b depicts an STM image of the HOPG substrate after Cp^*^Ru^+^ fragment adsorption at a bias voltage of -20 mV and a set point current of 150 pA. In the STM images, low-height hazy and particle-like protrusions were observed. We assumed that the low-height hazy protrusions indicate contamination on the surface induced by the solution dipping process of the HOPG substrate. Meanwhile, the bright particle-like protrusions were identified as the Cp^*^Ru^+^ fragment bound on the HOPG surface, with diameters of 6.41 ± 0.79 Å. These dimensions were similar to the calculated lateral size (7.2 Å) of Cp^*^ considering the van der Waals radius of the atoms, although differed slightly in other aspects. This might be because the STM data were based on the measurement of the tunneling current between the metal tip and surface rather than van der Waals interactions. In addition to the presence of Cp^*^Ru^+^ fragments on the surface, we observed uneven bending of the HOPG surface after fragment adsorption in the STM images, as shown in Figures S5, S6, and S7. The most reasonable explanation for this morphological change is the redevelopment of the graphene surface for minimizing the system energy, which was induced by the increase in the compressive surface stress based on the adsorption of Cp^*^Ru^+^ fragments. To corroborate the observation in Fig. 2b, the minimum energy configuration of Cp^*^Ru^+^-graphene was calculated via a simulation of the molecular mechanics force field, as shown in Figure S6. The results confirmed that the honeycomb structures of the graphene surface bound with the Cp^*^Ru^+^ fragment were concavely bent as the calculation progressed, thereby corresponding to the STM image of the HOPG surface bound with Cp^*^Ru^+^ fragments. Discussions regarding the minimum energy configuration of Cp^*^Ru^+^-graphene will be provided in a later section.

**Figure 3:**
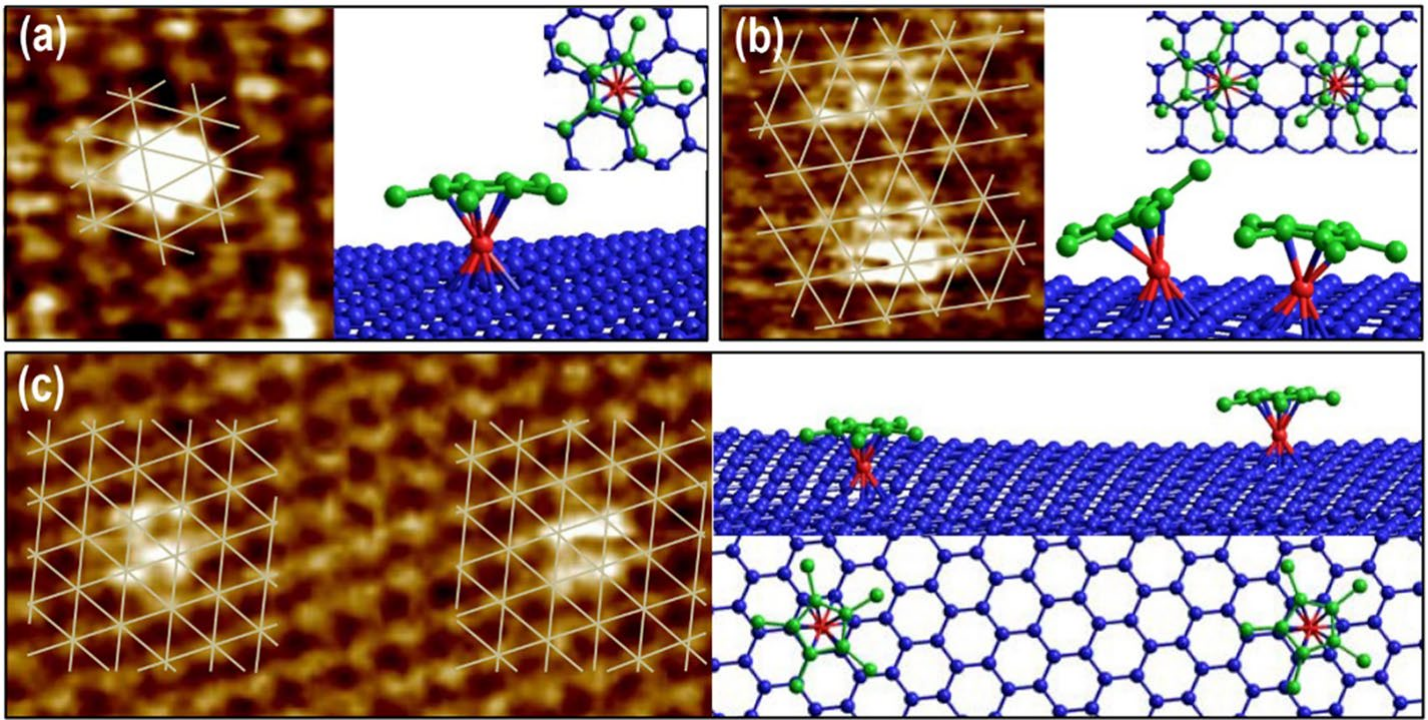
(**a**–**c**) Detailed STM images of Cp^*^Ru^+^ fragment bound variously on graphite lattice structure (left image) and their geometric configurations (right image). Each geometric configuration of Cp^*^Ru^+^ fragment on HOPG substrate show lowest energy states calculated from molecular mechanics force field. Hexagonal meshes shown in STM images depict graphite lattice structure comprising *β*-site carbons located above hollow site. Note that only one layer of the graphite was represented to clearly express the adsorbed position in the geometric configuration of Cp^*^Ru^+^-graphite on the right of each figure.

For a more detailed analysis of the Cp^*^Ru^+^ fragment adsorbed on the graphene surface, atomically resolved STM images of Cp^*^Ru^+^-graphene were magnified and sketched in a mesh with crossing points indicating the position of the *β*-site carbon in the honeycomb structure, as shown in Fig. 2. Figure 3a shows a single Cp^*^Ru^+^ fragment bound on the HOPG surface, which is represented as a bright protrusion with a lateral size of 6.2 Å. In principle, the dark spots in the STM image of graphite can either be the lattice site of an *α*-site carbon or a hollow position in the honeycomb structure^39^. By analyzing the height relations among the *α*-, *β*-, and hollow sites, these types of sites can be determined^40^. As shown in Fig. 3a, the center of the Cp^*^Ru^+^ fragment was above the hollow position of the carbon hexagon structure, as shown in Figure S6. Two Cp^*^Ru^+^ fragments with round and elliptical shapes that were bound to the neighboring hollow sites in the honeycomb are shown in Fig. 3b. This result can be understood from the simulated geometric configurations of the Cp^*^Ru^+^-graphene with the lowest energy via a simulation of the molecular mechanics force field. Based on geometric calculations and mesh visualization, the distance between hexagon centers bound with Cp^*^Ru^+^ fragments was 4.4 Å, which was similar to the molecular lateral size of the Cp^*^ fragment. Closely located neighboring fragments might result in a repulsion force between each fragment due to the steric effect; therefore, the top-view geometry of one fragment can be slanted and exhibit an elliptical shape, as shown in the configuration in Fig. 3b. Meanwhile, Fig. 3c shows two Cp^*^Ru^+^ fragments with sufficient interfragment distance on the HOPG (or graphene) surface. In this case, the fragments were round, indicating parallel Cp^*^ along the surface; this was observed because a repulsion force did not occur between the fragments owing the sufficient distance between them. This result is supported by the calculated geometric configuration with the lowest energy, as shown in the right image of Fig. 3c.

**Figure 4:**
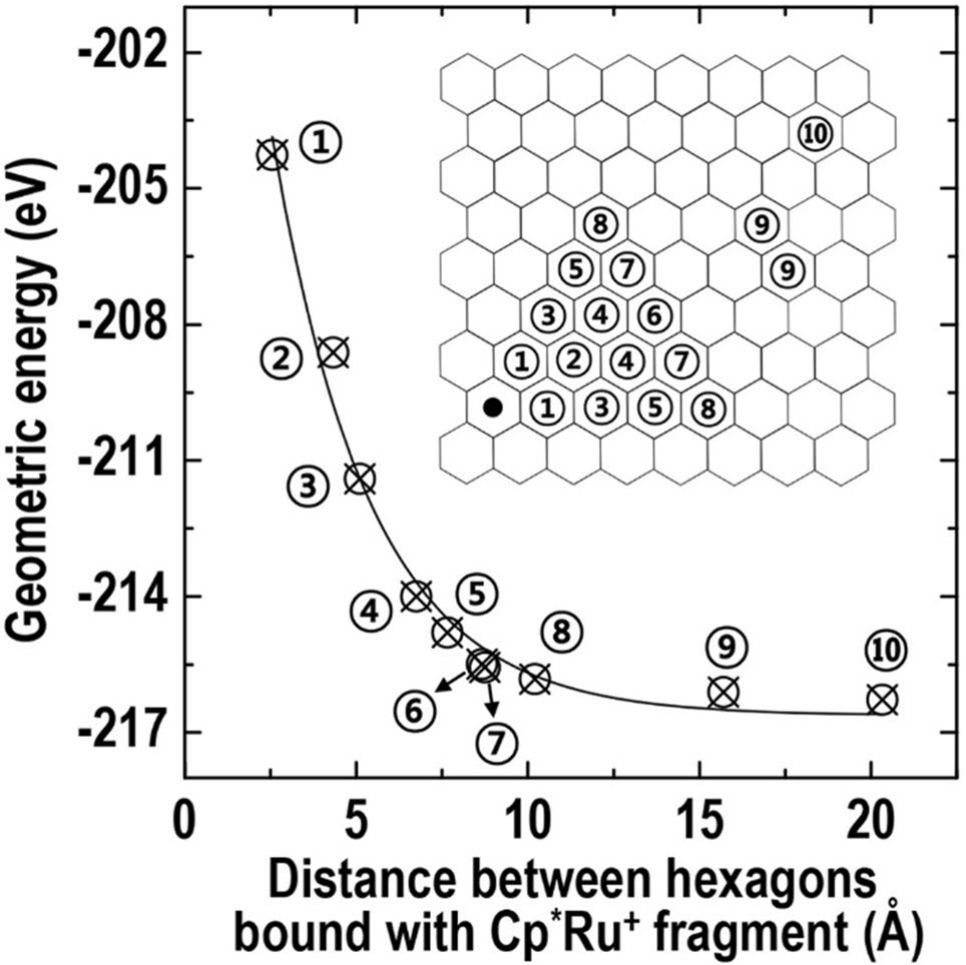
Simulated geometric energies of Cp^*^Ru^+^-graphene anchored with two fragments as a function of distance between hexagons bound with the fragment.

To understand the morphological deformation of the graphene surface, we simulated the geometric energy variation of the Cp^*^Ru^+^-graphene system as a function of the distance between Cp^*^Ru^+^ fragments anchored with hexagons, as shown in Fig. 4. In our calculation, the geometric energy of Cp^*^Ru^+^-graphene included both the energy variation for the morphological deformation of the graphene surface and that for the anchored geometry of the Cp^*^Ru^+^ fragments. This curve demonstrated that the geometric energy was associated significantly with the distance between the Cp^*^Ru^+^ fragments, and that it increased considerably in less than 7.65 Å (case ⑤), which is similar to the lateral size (7.2 Å) of Cp^*^. Therefore, we assumed that the increase in the geometric system energy can be induced primarily by the steric interaction force between the adsorbed fragments. This assumption can facilitate the understanding of the geometric configuration of Cp^*^Ru^+^-graphene based on the anchoring distance between the Cp^*^Ru^+^ fragments.

In addition, the analysis of an adsorption site and a geometric configuration of Cp^*^Ru^+^-graphene indicates the presence of strong *η*^6^-binding interactions between Cp^*^Ru^+^ and the hexagonal structure on the graphene by inducing a 6π-electron donor. In the STM results shown in Fig. 3b, we observed not only the presence of the Cp^*^Ru^+^ fragment on the graphene lattice, but also the slantly deformed Cp^*^Ru^+^ geometry by the closely located neighboring fragment. If the binding force of the Cp^*^Ru^+^ fragment on graphene is weak, then these results will not be obtained owing to the desorption and movement of fragments. Such behaviors on the surface would result in fuzzy STM images and would not maintain the increased geometric energy by the structural deformation of the Cp^*^Ru^+^-graphene. For instance, the Cp^*^Ru^+^-graphene shown in Fig. 3b was calculated to have an energy of − 208.6 eV, which is 7.5 eV more unstable than that shown in Fig. 3c. This energy difference implies that the Cp^*^Ru^+^-graphene with a weak binding force between the Cp^*^Ru^+^ fragment and the arene structure cannot maintain its structure.

**Figure 5:**
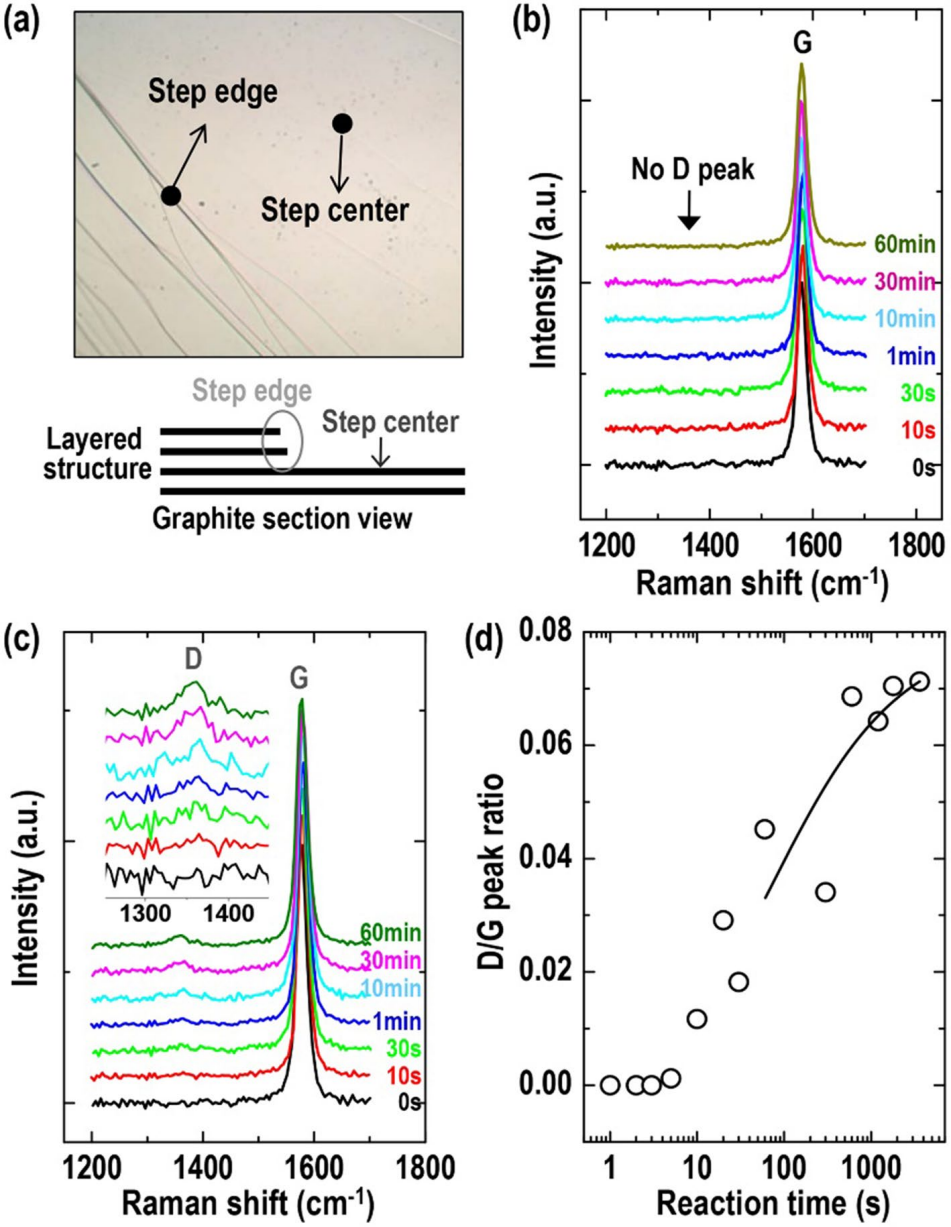
(**a**) Optical microscopic image and schematic section view of HOPG surface with a graphene-layered structure. Numerous graphite step centers and edges are formed during mechanical exfoliation. (**b**) *G* and *D* peak Raman spectra on the step center of graphite as a function of reaction time in 1.25 × 10^−7^ M Cp^*^RuL_3_PF_6_ solution. (**c**) *G* and *D* peak Raman spectra and (**d**) *D*/*G* peak intensity ratio on step edge of graphite as a function of reaction time in 1.25 × 10^−7^ M of Cp^*^RuL_3_PF_6_ solution. Inset of panel (**c**) shows enlarged *D* peak region of Raman spectra.

To consider the possibility of defect formation on the graphene (or HOPG) surface during the adsorption of Cp^*^Ru^+^ fragments, we measured the Raman spectra at both the center and step edge of the HOPG surface shown in Fig. 5a as a function of reaction time. Typically, two bands appear in this range of Raman shift: the *D* band (~ 1350 cm^−1^) and *G* band (~ 1580 cm^−1^)^41^. The graphite Raman *D* band provides evidence of the presence of intrinsic defects that disrupt the π-conjugation and convert sp^2^ carbon atoms to sp^3^ carbon atoms. Therefore, no *D* band on the HOPG indicates a high-quality substrate that is free of defects. Figure 5b shows the resultant Raman spectra at the center of the HOPG surface, and the *D* band was not observed. This finding is typical for mechanically exfoliated HOPG samples^42^. Upon reacting Cp^*^Ru^+^(CH_3_CN)_3_ on the surface, the *D* band did not evolve in the spectra with the reaction times. This result indicates that the adsorbed Cp^*^Ru^+^ fragments could not derive the intrinsic or acquired defects, although they caused the morphologically uneven deformation of the graphene surface. However, on the step edge, the Raman *D* band evolved as the reaction time progressed, as shown in Fig. 5c. To quantitatively analyze the defect level, we analyzed the Raman *D*/*G* peak ratio related to the defect density, as shown in Fig. 5d. As shown in the results, the *D*/*G* peak ratio increased gradually from zero to 0.074 as the reaction time progressed, although the ratio was extremely small compared with those reported the literature^42^. Subsequently, we investigated the origin of the *D* peak. The *D* peak was absent on both the step edge of the pristine HOPG case and the step center of the HOPG case bound to the Cp^*^Ru^+^ fragments. Therefore, the adsorption of the fragments above the hexagonal structure on the step edge did not contribute to the intrinsic defects on the graphene, despite the increase in the *D*/*G* peak ratio. To infer the origin of the *D* peak evolution on the step edge of the HOPG surface, we analyzed the C1s core level region of the pristine HOPG surface based on the XPS spectra shown in Figure S8. The C1s peak was composed of combinations of other peaks related to oxidation and can be deconvoluted into sp^2^-hybridized C–C in the aromatic ring (284.6 eV), C–O (286.2 eV), and C=O (287.3 eV)^42,43^, although freshly exfoliated HOPG substrates were used in the XPS measurements. (In this case, O=C–O contributions (289.1 eV) could not be extracted from the C 1s peaks because of the insignificant contributions.) The presence of oxygen-related carbon peaks is expected because oxygen molecules easily react with the dangling bonds at the step edge^44^. Therefore, we assumed that the hydroxyl and carbonyl groups at the edge can result in additional reactions with Ru^+^ in [Cp^*^Ru(CH_3_CN)_3_]^+^, inducing sp^3^ carbon structures in the honeycomb structure on graphite. Hence, the *D* peak evolved at the step edge by binding to the fragments, as shown in Fig. 5d. However, this is merely our speculation; further studies are necessitated to identify the exact reason.

## Conclusions

In summary, we demonstrated the adsorption behavior and geometric configuration of Cp^*^Ru^+^ fragments on a HOPG surface with a highly ordered arene nanostructure using STM measurements and calculations. Our results showed that a Cp^*^Ru^+^ fragment was localized above the hollow position of the hexagonal carbon structure in the STM images, and that the HOPG surface adsorbed with the fragments was morphologically redeveloped by minimizing the geometric energy. In particular, by calculating the geometric energy variation, we discovered that the system geometric energy of Cp^*^Ru^+^-graphite increased significantly at distances less than 7.65 Å between the Cp^*^Ru^+^ fragments, as a result of the steric effect of Cp^*^. These findings indicated that a combination of geometric configuration calculations and experimental studies can provide valuable insight into the behavior of adsorbed molecules for the identification of geometric characteristics and eventual design of more effective organometallic complexes.

## Supplementary Information

Supplementary Information.
