# Supplementary material for: Understanding adsorption geometry of organometallic molecules on graphite
Source: Sci Rep. 2021 Sep 16;11:18497. doi: 10.1038/s41598-021-97978-x (PMC8446079; doi:10.1038/s41598-021-97978-x)
Supplement: Supplementary file 1 — Supplementary Information. [file 41598_2021_97978_MOESM1_ESM.docx]

**Supporting information**

for

**Understanding Adsorption Geometry of Organometallic Molecules on Graphite**

*Seungtaek Oh^1,2^, Jungyoon Seo^1,2^, Giheon Choi^1,2^, Hwa Sung Lee^1,2*^*

^1^Department of Materials Science and Chemical Engineering, Hanyang University, Ansan Gyeonggi 15588, Republic of Korea.

^2^BK21 FOUR ERICA-ACE Center, Hanyang University, Ansan, Gyeonggi 15588, Republic of Korea

*Corresponding author. E-mail: hslee78@hanyang.ac.kr (H. S. Lee)


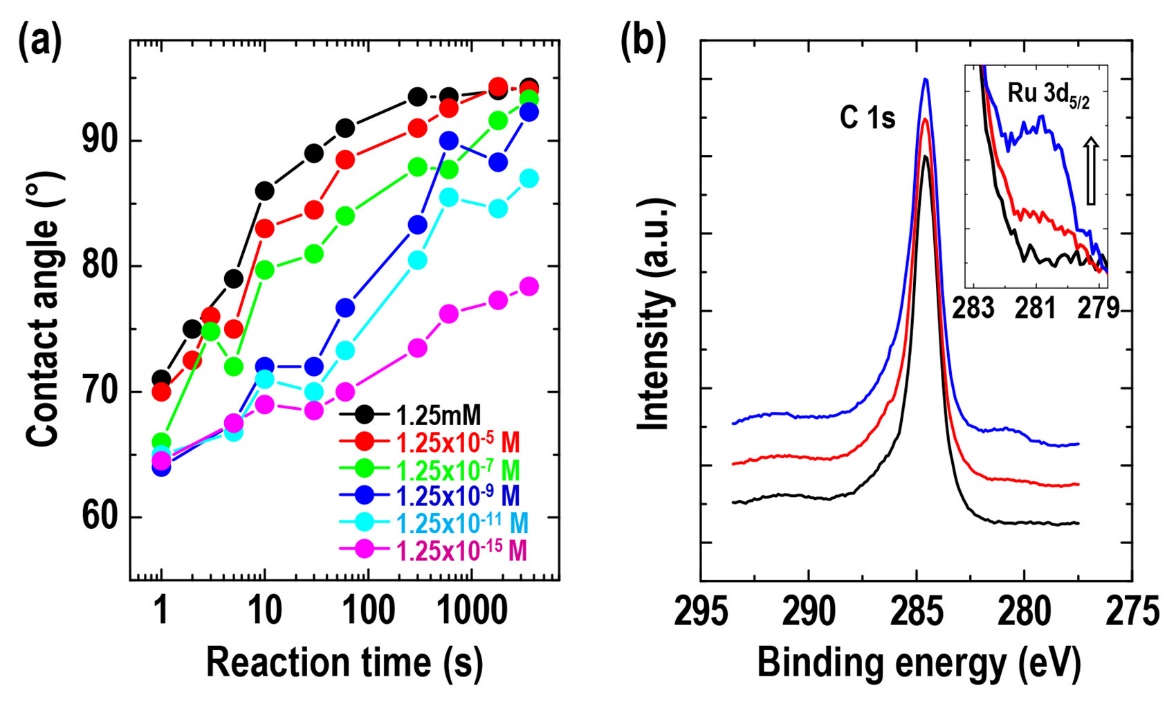


**Figure S1. (a)** Contact angle variations of deionized water on graphite surface as functions of reaction time and concentration of [Cp^*^Ru(CH_3_CN)_3_]^+^ solutions. Contact angle increased with reaction time and solution concentration, i.e., from 60° for pristine to 95° for HOPG reacted with 1.25 mM of [Cp^*^Ru(CH_3_CN)_3_]^+^ solution for 1 h. This increase is attributable to the decrease in surface energy of reacted graphite induced by five methyl groups in Cp^*^. **(b)** XPS spectra of Ru 3d_5/2_ signal regions for pristine graphite (black) and Cp^*^Ru^+^-graphites reacted with 1.25×10^-7^ M (gray) and 1.25 mM (dark gray) solutions.


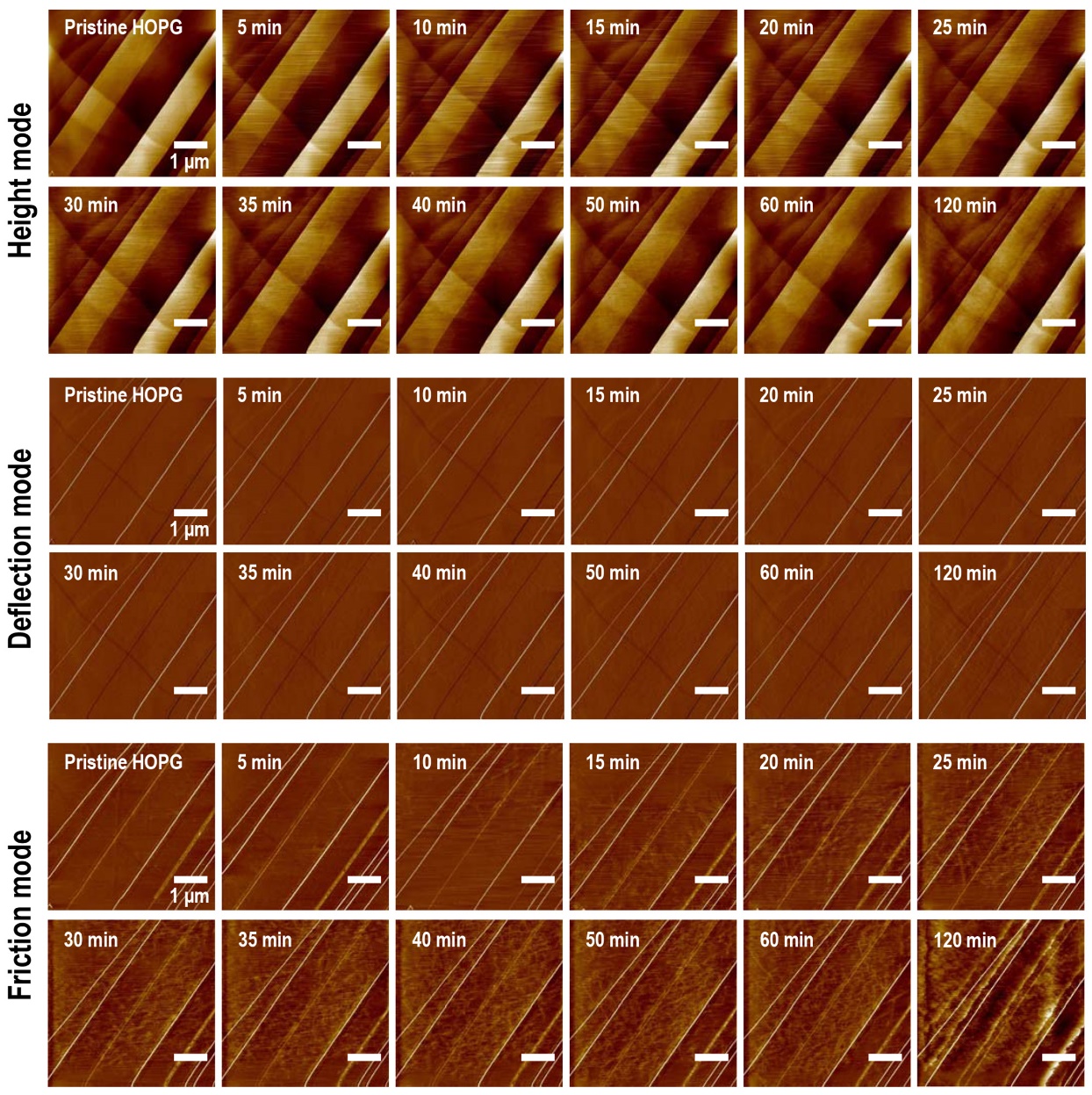


**Figure S2**. Sequential images of HOPG surface obtained via real-time in-situ AFM during reaction in 1.25 × 10^-11^ M of Cp^*^Ru(CH_3_CN)_3_PF_6_ solution. In height and deflection modes of AFM, morphological evolutions on surface were barely visible, likely due to small molecular size (approximately 3.5 Å in height and approximately 5 Å in width) of Cp^*^Ru^+^ fragment. By contrast, friction mode shows consecutive modification of HOPG surface by adsorbing Cp^*^Ru^+^ fragments. This occurred because surface energy was reduced by methyl groups of Cp^*^Ru^+^ fragment, thereby inducing a decrease in friction force between AFM tip and HOPG surface. Furthermore, adsorption of Cp^*^Ru^+^ fragments occurred evenly on the surface, not on specific regions such as cracks and steps of graphite surface. All scales are 1 μm in size.


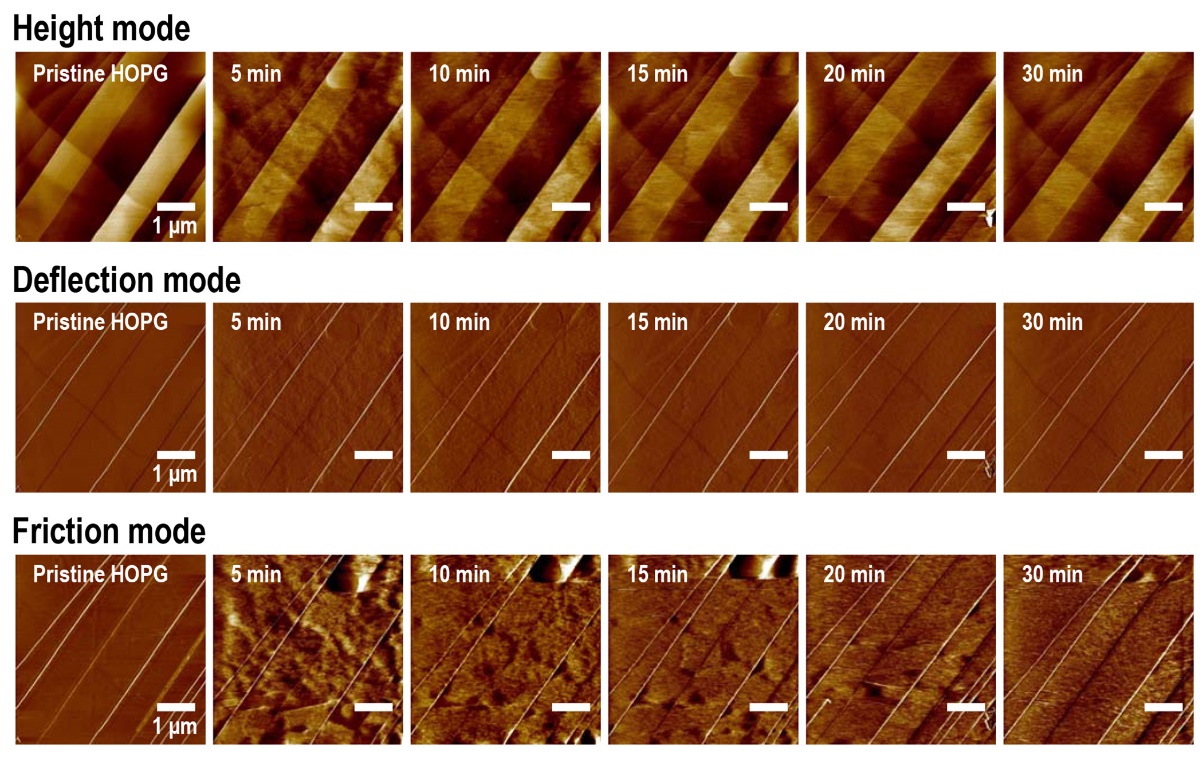


**Figure S3**. Sequential images of HOPG surface obtained via real-time in-situ AFM during reaction with 1.25 mM of Cp^*^Ru(CH_3_CN)_3_PF_6_ solution. All scales are 1 μm in size.


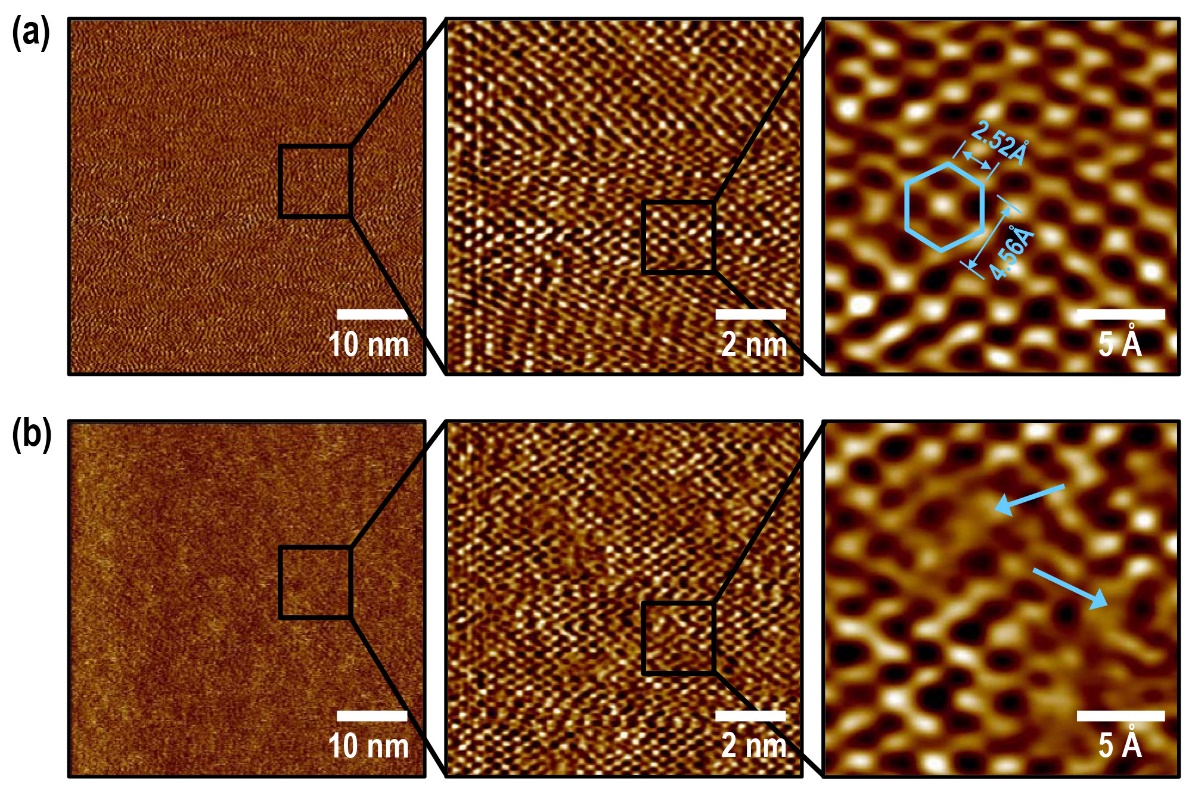


**Figure S4**. AFM images of **(a)** pristine graphite and **(b)** Cp^*^Ru^+^-graphite reacted in 1.25 × 10^-11^ M solution. In pristine graphite, hexagonal lattice structure of graphite surface without adsorbates was defined through magnified view. We observed many adsorbates (arrow regions) on Cp^*^Ru^+^-graphite surface with weak topographic contrast; therefore, the shapes and adsorbed positions on graphite lattice structures could not be clarified. We conclude that AFM measurements for obtaining clear molecule images of Cp^*^Ru^+^ fragments are impossible due to AFM resolution limitation despite operating under liquid ambience and the extremely small Cp^*^Ru^+^ fragments.


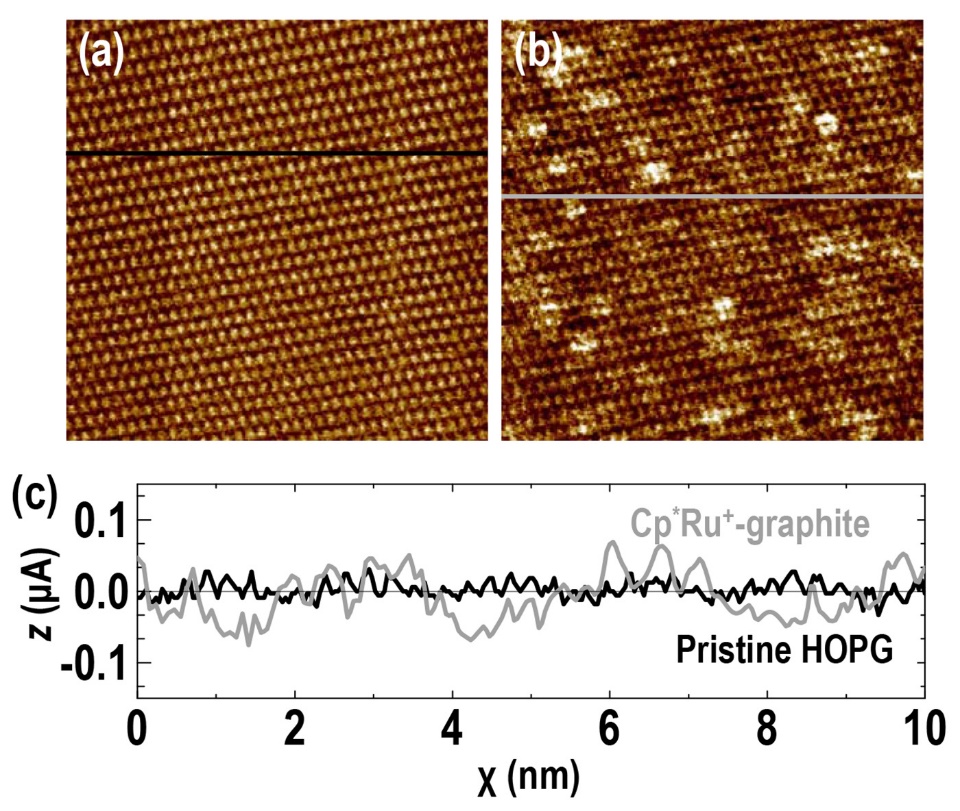


**Figure S5**. STM images of **(a)** pristine and (**b)** Cp^*^Ru^+^-graphites. **(c)** shows section profiles shown in **(a)** (black) and **(b)** (gray).


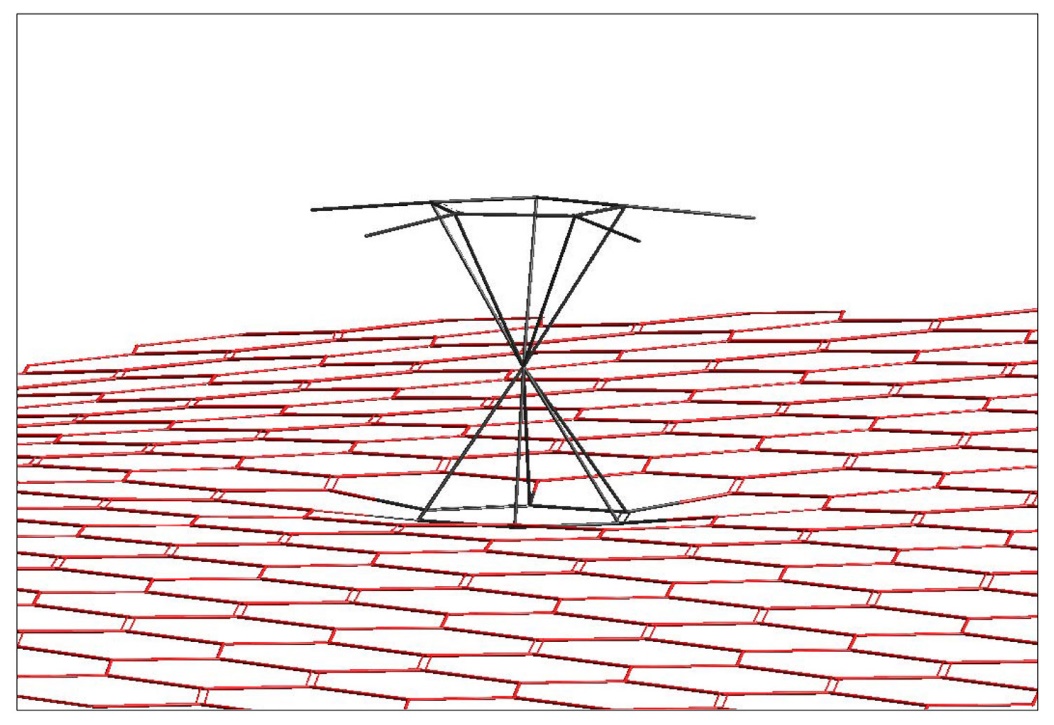


**Figure S6**. Minimum energy configuration of Cp^*^Ru^+^-graphene calculated via molecular mechanics force field. A concavely bent graphite structure bound with a Cp^*^Ru^+^ fragment, which is induced by the increase in compressive surface stress due to adsorption of fragment, is shown.


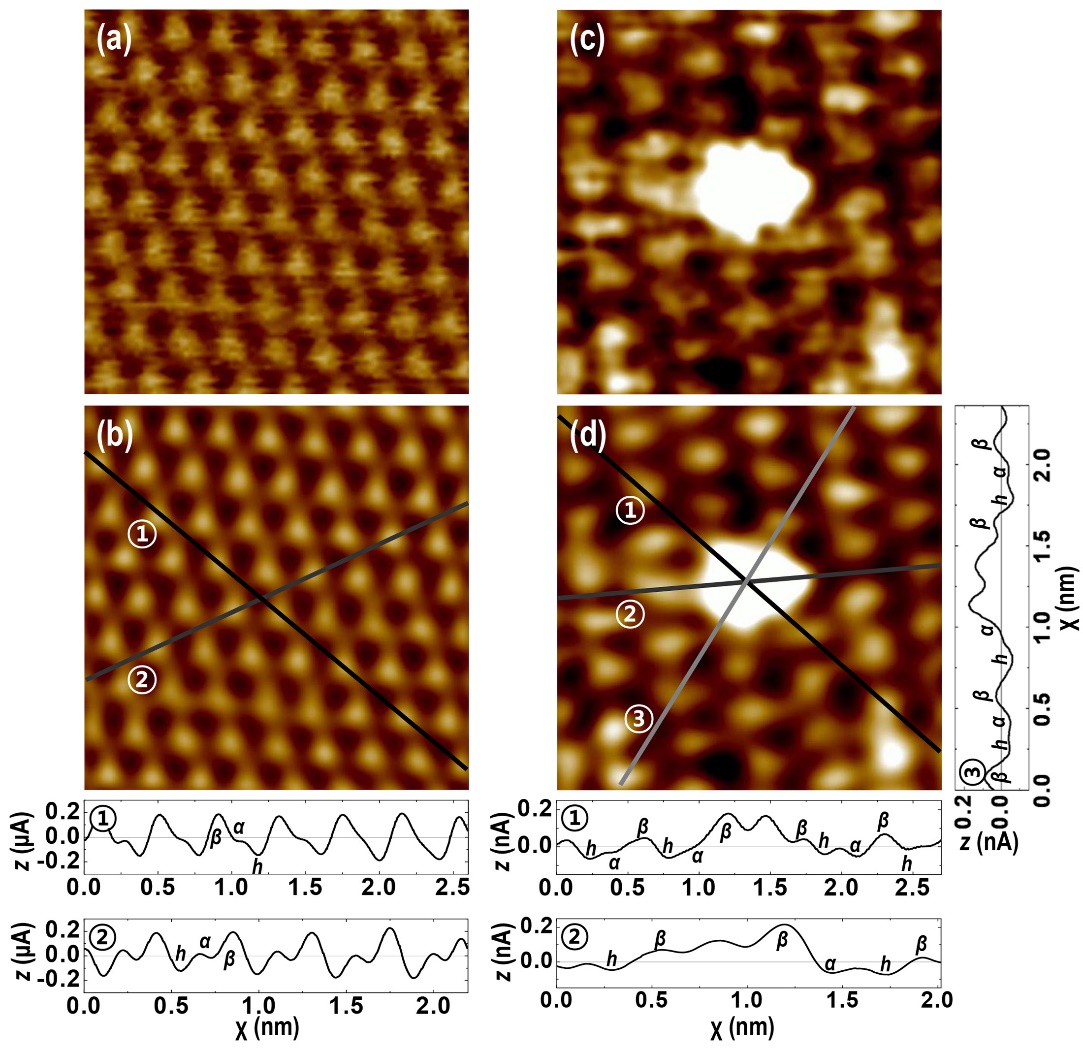


**Figure S7**. STM lattice images of **(a)** HOPG and **(c)** Cp^*^Ru^+^ fragment absorbed on surface. **(b)** and **(d)** show Fourier-filtered data and cross-sections of **(a)** and **(c)**, respectively. Two peaks are shown in cross section of STM image. They exhibit current relations between α- and β-site carbons and hollow sites resulting from their charge density asymmetry. According to imaging mechanism theory,^1-4^ bright spots with higher intensity correspond to β-site carbons and spots with less intensity to α-site. Hence, we conclude that absorbing site of Cp*Ru+ fragment is above hollow position of hexagon structure of graphite surface.


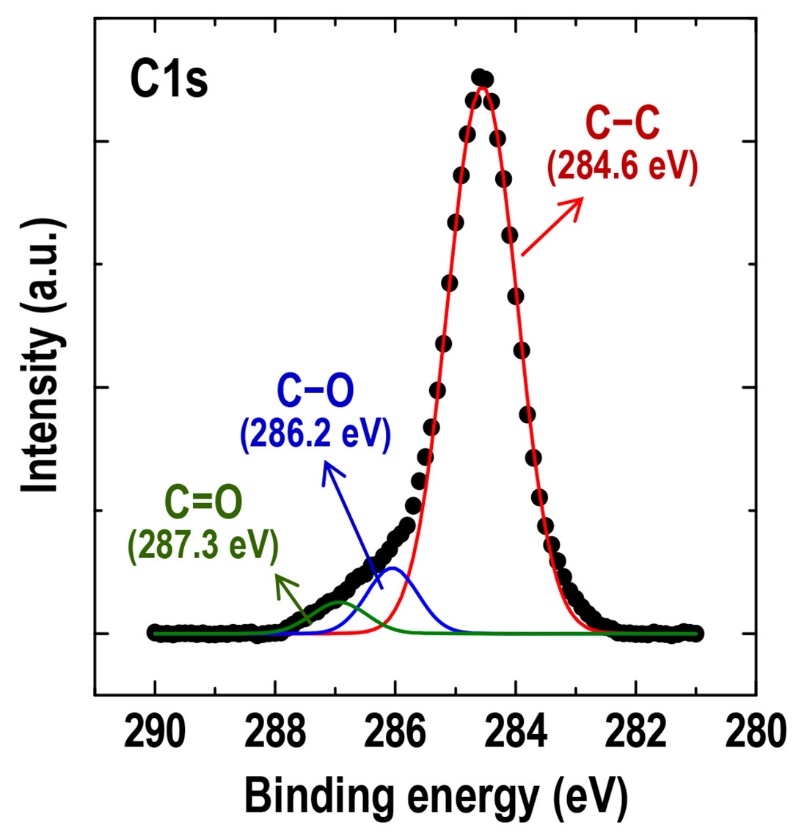


**Figure S8**. XPS spectra and fit of the C1s core level region of pristine HOPG.

**References**

1. Atamny, F.; Spillecke, O.; Schlögl, R. On the STM Imaging Contrast of Graphite: Towards a “true’’ Atomic Resolution. *Phys. Chem. Chem. Phys*. **1999**, *1*, 4113.
2. Hembacher, S.; Giessibl, F. J.; Mannhart, Local Spectroscopy and Atomic Imaging of Tunneling Current, Forces, and Dissipation on Graphite. J. *Phys. Rev. Lett*. **2005**, *94*. 056101.
3. Tománek, D.; Louie, S. G. First-Principles Calculation of Highly Asymmetric Structure in Scanning-Tunneling-Microscopy Images of Graphite. *Phys. Rev. B* **1988**, *37*, 8327.
4. Hembacher, S.; Giessibl, F. J.; Mannhart, J.; Quate, C. F. Revealing the Hidden Atom in Graphite by Low-Temperature Atomic Force Microscopy. *PNAS*, **2003**, *100*, 12539.
